# RAB21 interacts with TMED10 and modulates its localization and abundance

**DOI:** 10.1242/bio.045336

**Published:** 2019-08-27

**Authors:** Tomas Del Olmo, Camille Lacarrière-Keïta, Caroline Normandin, Dominique Jean, François-Michel Boisvert, Steve Jean

**Affiliations:** Faculté de Médecine et des Sciences de la Santé, Département d'anatomie et de biologie cellulaire, Université de Sherbrooke, 3201, Rue Jean Mignault, Sherbrooke, Québec, Canada J1E 4K8

**Keywords:** Membrane trafficking, TMED2, TMED9, p24 family

## Abstract

Membrane trafficking controls vesicular transport of cargo between cellular compartments. Vesicular trafficking is essential for cellular homeostasis and dysfunctional trafficking is linked to several pathologies such as neurodegenerative diseases. Following endocytosis, early endosomes act as sorting stations of internalized materials, routing cargo toward various fates. One important class of membrane trafficking regulators are RAB GTPases. RAB21 has been associated with multiple functions and regulates integrin internalization, endosomal sorting of specific clathrin-independent cargo and autophagy. Although RAB21 is mostly associated with early endosomes, it has been shown to mediate a specific sorting event at the Golgi. From mass spectrometry data, we identified a GTP-favored interaction between RAB21 and TMED10 and 9, essential regulators of COPI and COPII vesicles. Using RAB21 knockout cells, we describe the role of RAB21 in modulating TMED10 Golgi localization. Taken together, our study suggests a new potential function of RAB21 in modulating TMED10 trafficking, with relevance to neurodegenerative disorders.

## Abstract (precis)

**Summary:** A small early endosomal RAB GTPase is found to interact with p24 family members, with potential impacts on p24 functions.

## INTRODUCTION

Membrane trafficking, which represents all vesicular exchanges between organelles and cellular compartments, is highly regulated and essential for cellular homeostasis (Vicinanza et al., 2008). Indeed, trafficking defects are involved in a large panel of diseases, such as neurological pathologies (Stenmark, 2009). One important class of membrane trafficking regulators are the RAB GTPases (Jean and Kiger, 2012). With almost 70 members, RABs represent the largest family of small GTPases in humans (Rojas et al., 2012). These proteins mediate each step of vesicular trafficking, from membrane budding to vesicle transport, to fusion with target organelles (Hutagalung and Novick, 2011). Given their roles in trafficking, RABs are tightly regulated (Barr and Lambright, 2010). Thus, RABs cycle between their active GTP-bound form and inactive GDP-bound form. RABs are activated by GEFs (guanine exchange factors), which catalyze the exchange of GDP to GTP and are inhibited by GAPs (GTPase-activated proteins) that trigger intrinsic hydrolytic activity of RABs (Barr and Lambright, 2010). Once activated, RABs recruit a large number of effectors to achieve their functions (Grosshans et al., 2006). Moreover, RABs can directly interact with cargo to regulate their trafficking (Pellinen et al., 2006).

RAB21 regulates integrin internalization by binding directly to α5β1 integrin (Pellinen et al., 2006). Initially described as an early endosomal RAB (Simpson et al., 2004), RAB21 has been shown to be involved in various specific functions. It mediates EGFR degradation (Yang et al., 2012), controls neurite extensions (Burgo et al., 2009, 2012), regulates autophagic flux (Jean et al., 2015) and was recently shown to be associated with clathrin-independent cargo trafficking via WASH and retromer complexes (Del Olmo et al., 2019). In the same study, potential interactions between TMED10, TMED9 and RAB21 were observed by quantitative mass spectrometry analysis (Del Olmo et al., 2019).

TMED10 and TMED9 both belong to the p24 family of proteins (Pastor-Cantizano et al., 2016). These proteins are mostly localized between the ER and Golgi compartments, cycling between both of them and mediating cargo transport through COPI and COPII vesicles (Popoff et al., 2011). All proteins of the p24 family are composed of a GOLD intraluminal domain allowing interaction with cargo (Anantharaman and Aravind, 2002), a coiled-coil domain, a transmembrane domain mediating homo- and hetero-dimerization (Contreras et al., 2012) and a short cytosolic domain involved in protein sorting (Contreras et al., 2004). The cytosolic domain has been demonstrated to interact with the GTP-bound ARF1 (Gommel et al., 1999), allowing recruitment of ERD2 and formation of COPI vesicles (Majoul et al., 2001). Importantly, TMED10 and TMED2 expression is necessary to maintain ER and Golgi integrity (Montesinos et al., 2012). RAB21 has been shown to sort VAMP7 at the Golgi (Burgo et al., 2012), and several members of the p24 family have been identified as potential RAB21-binding proteins by mass spectrometry analysis (Del Olmo et al., 2019). Therefore, we characterized the TMED10 and RAB21 interaction using biochemical and genetic approaches. This allowed us to define a RAB21 requirement for appropriate TMED10 localization and protein abundance.

## RESULTS

### TMED10 interacts indirectly with RAB21

Although the functional association of TMED10 with ARF1 has been well characterized (D'Souza-Schorey and Chavrier, 2006), potential interactions with RAB family GTPases have only been shown by proteomic analysis (Hein et al., 2015) or genetic screens (Blomen et al., 2015), but have not been further assessed. Our recent mass spectrometry data identified a potential interaction between RAB21, TMED10 and TMED9. Strong enrichment of these two proteins with wild-type or the GTP-bound form of RAB21 have been observed using quantitative interactomics experiments (Del Olmo et al., 2019), in both HeLa and HCT116 cells (see fig. S1A from Del Olmo et al., 2019).

**Fig. 1.**
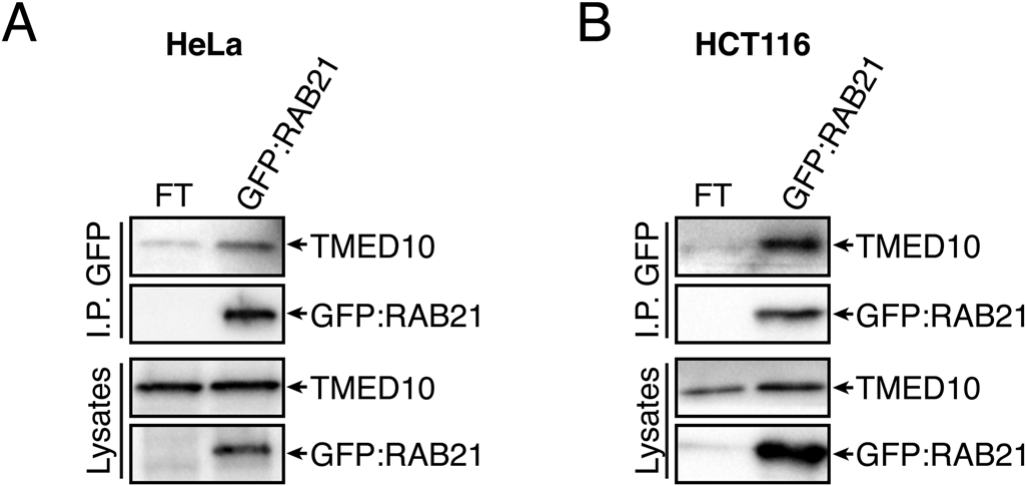
**RAB21 interacts with TMED10.** (A,B) Western blotting showing GFP-trap immunoprecipitation in HeLa (A) and HCT116 (B) cells. Endogenous TMED10 was blotted and showed a specific enrichment with GFP:RAB21-WT compared to Flp-In/T-REx (FT) control in both cell lines. Lysates represent 2% of input and *n*=4 independent experiments performed in each cell line.

To validate the interaction between RAB21 and TMED10, GFP co-immunoprecipitation assays were performed in Flp-In/T-REx HeLa and HCT116 cell lines that express GFP:RAB21 close to endogenous levels in response to doxycycline treatment, as described previously (Del Olmo et al., 2019). Consistent with the proteomics data, endogenous TMED10 was enriched in GFP:RAB21 immunoprecipitations in both HeLa (Fig. 1A) and HCT116 (Fig. 1B) cells, while an unrelated golgi protein, TGN38, was not (Fig. S1B). To test whether the identified interaction was direct, GST-pulldown assays were performed using purified GST:RAB21 or GST:RAB21-Q78L (GTP-bound) and incubated with HeLa cell lysates. Pulldown assays showed no specific enrichment of TMED10 or TMED2 with either wild-type or GTP-bound forms, although VPS35 (Del Olmo et al., 2019) was present with GST:RAB21 (Fig. S1C,D). From these results, we conclude that RAB21 interacts indirectly with TMED10.

### TMED10 interacts preferentially with activated RAB21

**Fig. 2.**
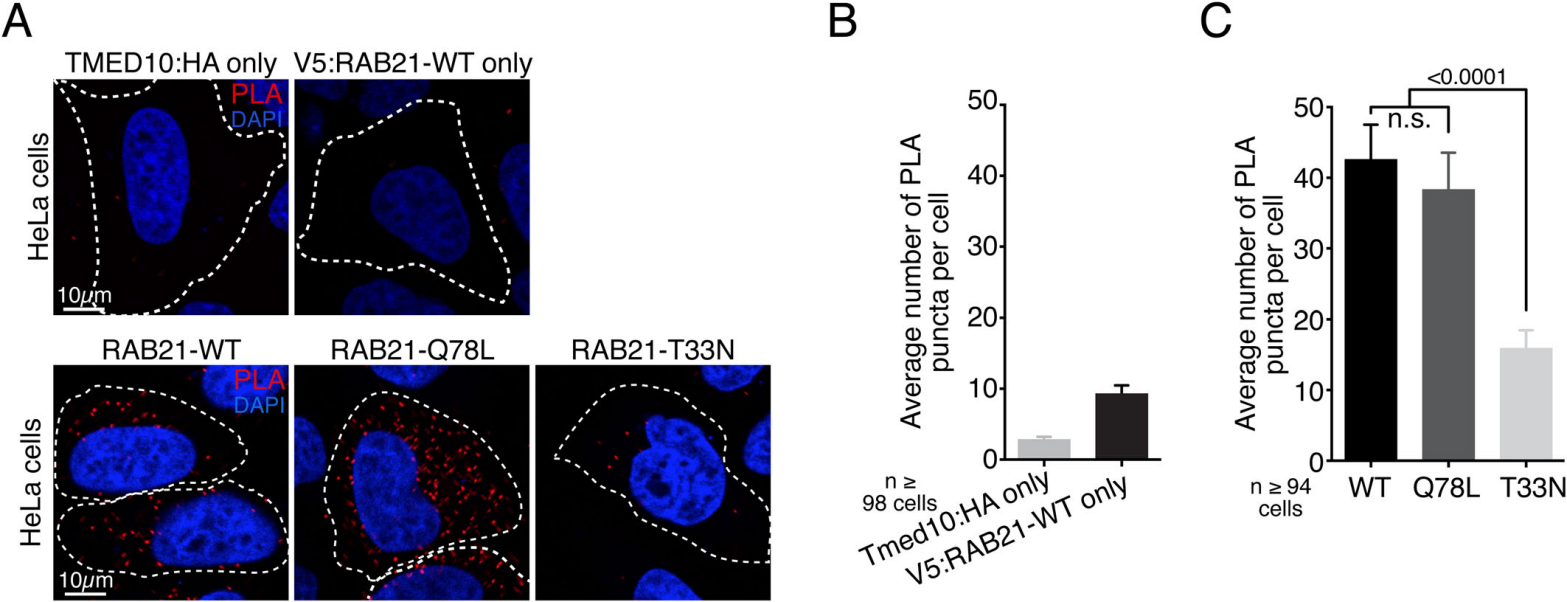
**RAB21 nucleotide status modulates the interaction with TMED10.** (A) PLA immunofluorescence showing respective proximities between TMED10:HA and either V5:RAB21 WT, Q78L or T33N variants. TMED10:HA and V5:RAB21 only are controls. PLA puncta are stained in red and nucleus in blue. Dotted lines define individual cells. Scale bars: 10 µm, *n*=2 independent experiments. (B) Quantification of PLA controls shown in A. Histogram represents average number of PLA puncta per cell, error bars are s.e.m. No statistical analysis was performed. (C) Quantification of PLA experiments shown in A. Histogram represents average number of PLA dots per cell in each RAB21 variant condition, error bars are s.e.m. Mann–Whitney tests were used for statistical analysis. n.s., not significant.

To assess the RAB21 interaction with TMED10, we performed proximity ligation assays (PLA). HeLa cells were singly or co-transfected with TMED10:3xHA and either V5:RAB21-WT, V5:RAB21-Q78L (GTP-bound) or V5:RAB21-T33N (GDP-bound) variants. The number of PLA puncta per cell (indicative of TMED10 and RAB21 proximity) was counted through confocal imaging and automated image analysis. While transfection of either TMED10 or RAB21 alone yielded a maximum of nine puncta per cell (Fig. 2A,B), co-transfection of TMED10 with RAB21-WT or RAB21-Q78L led to a considerable increase in the number of PLA puncta per cell, reaching an average of 42 puncta per cell in RAB21-WT cells. Notably, the number of PLA puncta per cell was significantly higher in RAB21-WT and RAB21-Q78L variants compared to RAB21-T33N (Fig. 2A,C). These PLA results are in accordance with the proteomics data and indicate that the interaction between RAB21 and TMED10 is increased upon RAB21 activation.

### RAB21 knockout affects TMED10 localization in cells

TMED10 has been reported to localize at the ER-Golgi interface. On the other hand, RAB21 localizes mostly on early endosomes except for the dominant negative RAB21 (T33N), which is strongly associated with the Golgi (Simpson et al., 2004). A previous study identified VARP- and RAB21-dependent functions in VAMP7 trafficking at the Golgi in neuronal cells (Burgo et al., 2012). Therefore, we assessed the functional relationship between RAB21 and TMED10 in RAB21 knockout cells. Phenotype specificity was ensured by generating two independent cell populations using two independent guide RNAs. These cells have previously been validated by sequencing and western blot analyses (Del Olmo et al., 2019).

**Fig. 3.**
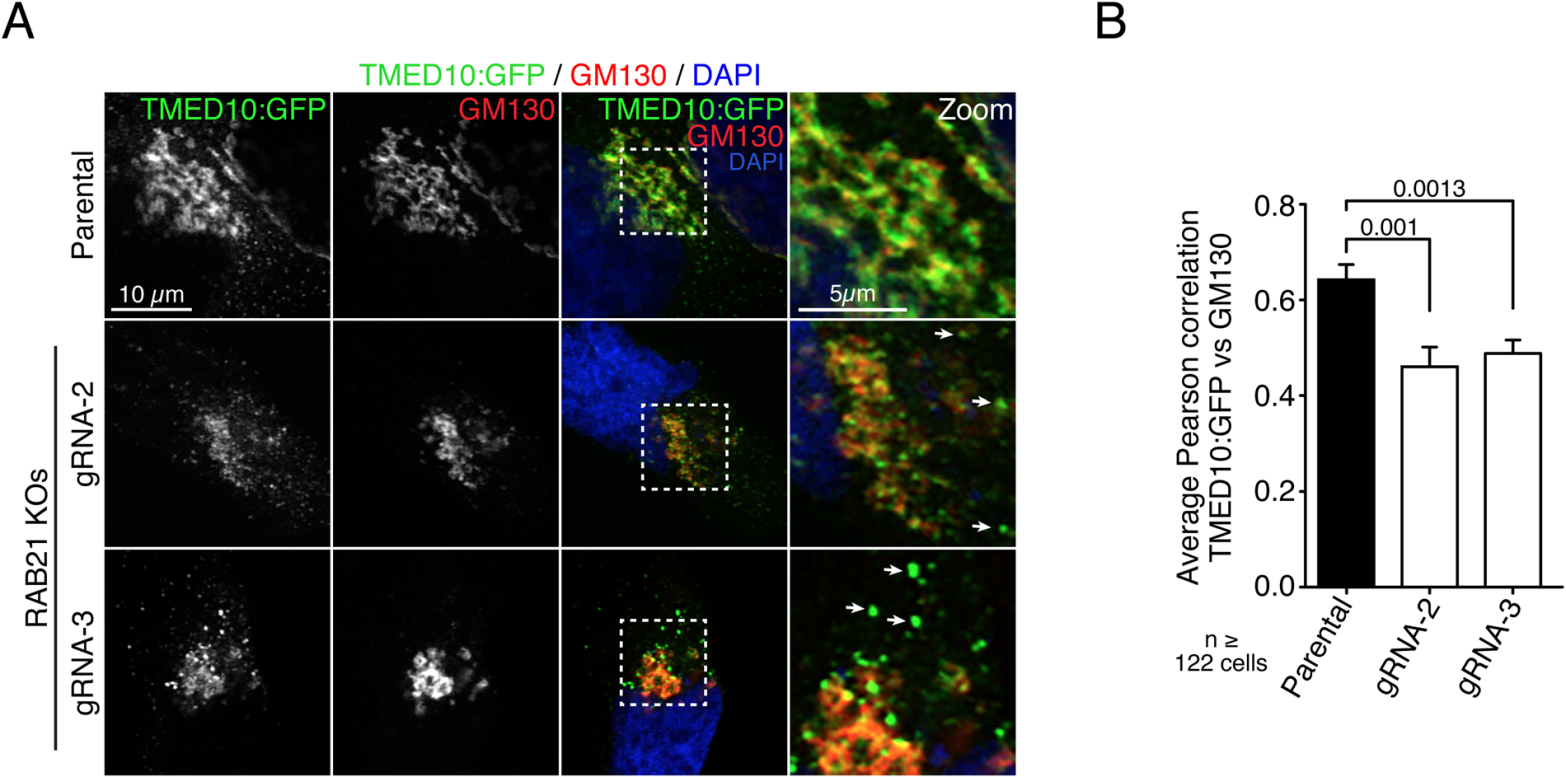
**TMED10 Golgi localization is altered in RAB21 knockout cells.** (A) TMED10:GFP colocalization with endogenous GM130 in parental and RAB21 knockout cells. gRNA-2 and -3 are two independent populations of RAB21 knockout HeLa cells. TMED10:GFP is stained in green, GM130 in red and nucleus in blue. Dotted squares are magnified on the right, arrows show TMED10:GFP-only-labeled vesicles. *n*=3 independent experiments. Scale bars: 10 µm; 5 µm in zoom. (B) Quantification of TMED10 and GM130 colocalization. Histogram represents average Pearson correlation per cell, error bars are s.e.m. Mann–Whitney tests were used for statistical analysis.

Parental and RAB21 knockout cells were transfected with GFP:TMED10 (Blum and Lepier, 2008) and TMED10 localization at *cis*- and *trans*-Golgi was investigated by colocalization with GM130, TGN46 and ci-MPR, respectively (Fig. 3A; Fig. S2A,B). Interestingly, RAB21 deletion reduced TMED10 localization in the *cis*-Golgi compartment (Fig. 3A). Multiple TMED10 puncta were observed outside the *cis*-Golgi in these cells. Moreover, TMED10 colocalization with GM130 significantly decreased in both HeLa-RAB21 KO cells compared to parental cells (Fig. 3B). TMED10 colocalization with TGN46 also decreased in both HeLa-RAB21-KO cell populations, however only the gRNA-2 population showed a statistical difference (Fig. S2C). Surprisingly, no difference was observed between TMED10 and ci-MPR colocalization (Fig. S2D). Given that ci-MPR also labels endocytic vesicles, an increased localization of TMED10:GFP in these vesicles could potentially compensate for the observed difference at the Golgi and yield similar Pearson correlation values. Taken together, these results suggest the role of RAB21 in TMED10 maintenance or targeting at the *cis*-Golgi compartment and potentially at the *trans*-Golgi as well.

### RAB21 depletion reduces TMED10 protein levels

**Fig. 4.**
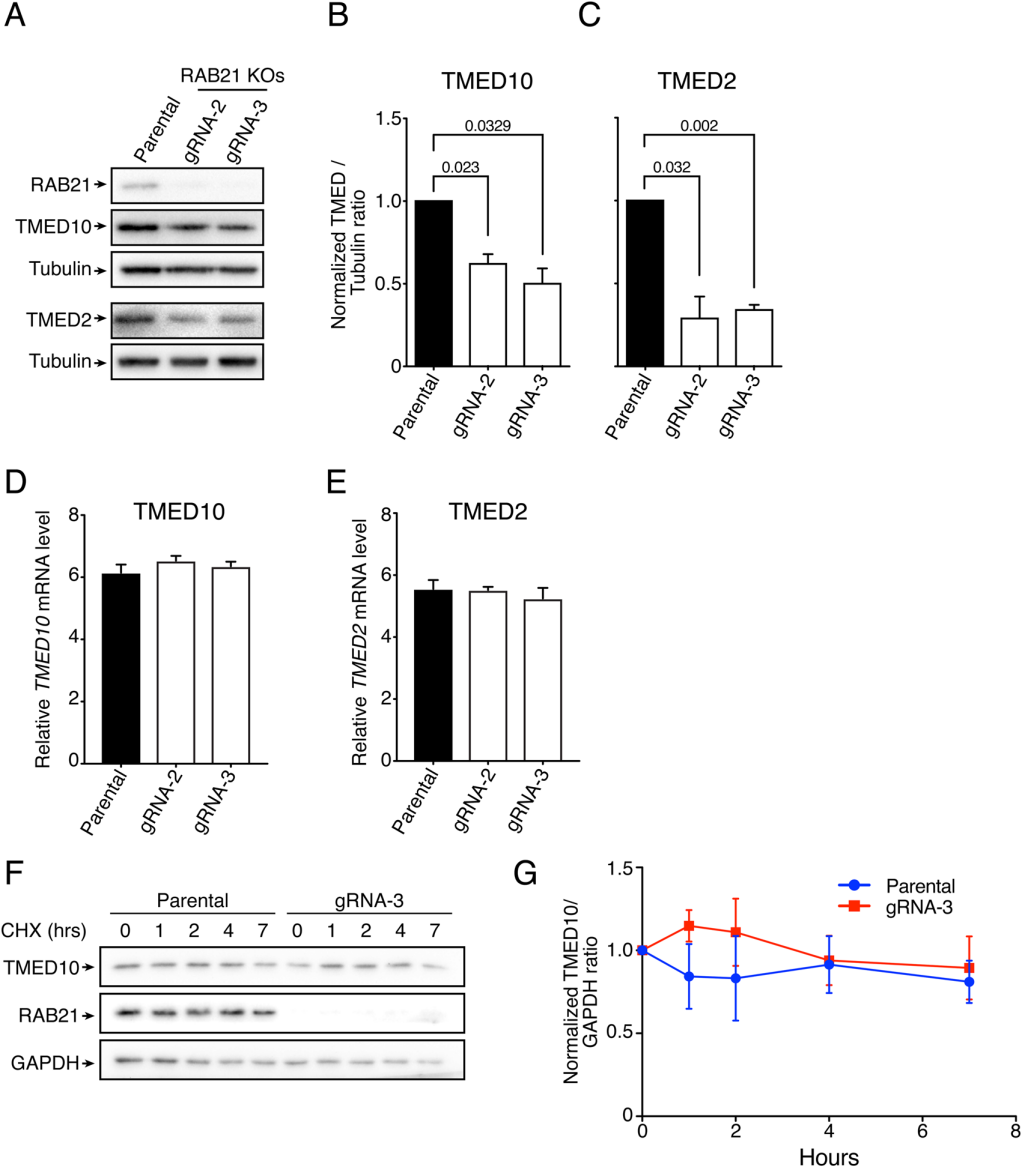
**RAB21 knockout reduces TMED10 and TMED2 protein levels.** (A) Western blotting showing TMED10 and TMED2 protein levels in parental and RAB21 knockout cells. Endogenous TMED10, TMED2, RAB21 and Tubulin were blotted. Tubulin was used as a housekeeping gene, *n*=4 independent experiments. (B,C) Quantification of relative protein expression shown in A. TMED10/Tubulin (B) and TMED2/Tubulin (C) protein ratios were normalized to parental cells, error bars are s.e.m. One-sample *t*-tests were used for statistical analysis. (D,E) Quantitative PCR analysis of TMED10 and TMED2 transcripts, respectively, *n*=4 independent experiments, error bars are s.e.m. Unpaired *t*-tests were used for statistical analysis. No statistical differences were observed and are thus not displayed. (F,G) TMED10 stability assay. Cycloheximide chases were performed to monitor TMED10 stability in parental and RAB21 gRNA-3 KO HeLa cells, *n*=3 independent experiments. (F) Endogenous TMED10, RAB21 and GAPDH were assessed through western blotting. (G) Quantification of relative protein expression shown in F, error bars are s.e.m. Unpaired *t*-tests were performed and no statistical differences were observed and therefore are not displayed.

Given that TMED10 was mis-localized in RAB21 knockout cells, we assessed whether TMED10 protein levels were also affected. Using western blotting, we compared relative TMED10 expression in parental and RAB21-KO HeLa cells (Fig. 4A). Relative protein quantification showed that in both RAB21 knockout populations, TMED10 expression was almost twice as low as in the control (Fig. 4B). Since p24 family members are known to oligomerize (Contreras et al., 2012) and depletion of TMED10 affects other p24 family members (Pastor-Cantizano et al., 2016), we also analyzed TMED2 protein abundance. In accordance with the result observed for TMED10, we found that TMED2 expression was also altered in RAB21 knockout cells (Fig. 4C). We assessed if this was due to changes in transcription or in protein stability. Quantitative PCR analyses of TMED2 and TMED10 did not highlight any significant difference in expression (Fig. 4D,E). Similarly, a cycloheximide chase did not show apparent differences in stability (Fig. 4F,G). We further assessed if TMED10 half-life was modulated by proteasome or lysosomal degradation. MG132 or Bafilomycin A1 treatments, which block proteasome or lysosome functions, respectively, did not significantly impact TMED10 protein levels in either parental or RAB21 knockout cells (Fig. S3A,B). From these results, we conclude that RAB21 is required for expression of TMED10 and TMED2, that TMED10 has a long half-life in HeLa cells and that the exact mechanism leading to TMED2 and 10 downregulation needs to be elucidated.

## DISCUSSION

First identified to play a specific role in integrin trafficking (Pellinen et al., 2006), RAB21 is now associated with several other functions (Del Olmo et al., 2019; Jean et al., 2015; Alanko et al., 2015). In the present study, we confirmed previous mass spectrometry data showing a potential interaction between RAB21 and TMED10 (Del Olmo et al., 2019). We conclude that activated RAB21 interacts indirectly with TMED10 as evidenced by immunoprecipitation analysis, proximity ligation assays and pull-down data. We further show that RAB21 is required for TMED10 localization in the Golgi and that RAB21 influences TMED10 and TMED2 protein expression.

TMED10 is mostly observed at the *cis*-Golgi (Pastor-Cantizano et al., 2016), compared to RAB21, which is mostly endosomal. However, RAB21 has been observed at the Golgi in neurons where it regulates, through VARP, the sorting of VAMP7 (Burgo et al., 2012). Hence, it is possible that another function of VARP would be regulation of RAB21 interaction with TMED10. The preferential interaction of TMED10 with activated RAB21 thus suggests that TMED10 could act as a RAB21 effector or that activated RAB21 could influence the interaction between TMED10 and its specific cargo. The data from RAB21 knockout cells indicates that both possibilities are plausible. However, it is unlikely that TMED10 would act as a RAB21 effector, due to the lack of a direct interaction between the two proteins. Hence, a Golgi-associated RAB21 pool could retain TMED10 at the Golgi by interacting with TMED10, possibly through an unknown protein. Alternately, RAB21 could strengthen or weaken TMED10 interaction with cargo, as observed for ARF1 (Luo et al., 2007) and RAB10 (Wang et al., 2010).

TMED10 has been observed to be localized at other intracellular compartments such as the ER, the ERGIC compartment, on secretory vesicles and at the plasma membrane (Pastor-Cantizano et al., 2016). Given the known functions of RAB21 in endocytosis and protein sorting (Jean et al., 2015; Pellinen et al., 2006; Del Olmo et al., 2019), and considering the observed RAB21-dependent TMED10 localization at the Golgi, it is also possible that RAB21 could be involved in TMED10 recycling from the plasma membrane to the Golgi. A recent study suggests that TMED10 cycles through the plasma membrane with improperly folded GPI-anchored proteins (Zavodszky and Hegde, 2019). Hence, an interesting possibility would be that RAB21 is involved in regulating the trafficking and degradation of improperly folded GPI-anchored proteins, and as such RAB21 depletion would lead to improper TMED10 localization.

In our recent proteomic study where we noticed RAB21/TMED10 interaction, we did not observe any significant enrichment of TMED10 in APEX2:RAB21-mediated proximity labeling. This was rather surprising given the large number of proximal RAB21 proteins identified in that study (Del Olmo et al., 2019). We believe that this could be explained by the fact that APEX2 biotinylation occurs mostly on tyrosine residues (Lee et al., 2017), and p24 family members contain only short cytosolic domains with no tyrosine (Pastor-Cantizano et al., 2016). Therefore, although TMED10 could still be proximal to APEX2:RAB21 in these experiments, it might not be biotinylated and hence detected. This indicates the necessity to combine experimental approaches to define protein interactomes. In this regard, both SILAC and proximity labeling approaches will complement each other in future studies.

RAB21 modulates TMED10 and TMED2 protein levels. TMED10 was found to have a half-life of 3 h in the neurons (Liu et al., 2008), while TMED2 was shown to have a very long half-life in Vero cells (Füllekrug et al., 1999). TMED10 degradation in neurons was mostly through the proteasome, while the mechanism in Vero cells has not been characterized. Hence, it remains unclear whether a common pathway is responsible for the degradation of p24 family members. From our data, we could not identify the mechanism which contributes to the decreased abundance of TMED10 and TMED2. We observed that TMED10 had a long half-life in HeLa (>7 h) and we could not detect a shorter half-life in RAB21 knockout cells. Furthermore, proteasome or lysosome inhibition for 16 h did not strongly affect TMED10 levels. We did observe a slight increase in TMED10 levels upon lysosome inhibition (Fig. S3B), but this observation was not consistent over the various repeats. Hence, decreased protein levels of TMED10 and 2 could be attributed to an indirect effect on other p24 family members or to a slight increase in degradation kinetics that could not be observed in the timescale of our experiment. We ruled out a general effect on global protein translation, since multiple proteins are not downregulated in RAB21 knockout cells (Del Olmo et al., 2019).

TMED10 plays a dual role in cargo trafficking. By itself, TMED10 is involved in specific secretion of GPI-anchored proteins (Theiler et al., 2014) or PAR-2 (Zhao et al., 2014) towards the plasma membrane. On the other hand, TMED10 has been shown to retain cargo such as MHC class I (Jun and Ahn, 2011) in the ER, and PKC-δ in Golgi-like structures (Wang et al., 2011), thus inhibiting their activities. Similarly, TMED10 is responsible for direct γ-secretase inhibition (Pardossi-Piquard et al., 2009). We hypothesized that RAB21 could modulate general TMED10-regulated cargo trafficking by disturbing TMED10 localization and stability. With respect to this hypothesis, RAB21 has been shown to interact with Presenilin 1, reducing APP synthesis through γ-secretase inhibition (Sun et al., 2017). Hence, it would be interesting to investigate if the involvement of RAB21 and TMED10 in γ-secretase inhibition are linked or independent. Moreover, inhibition of TMED10 expression is involved in ATG4-mediated autophagy activation (Shin et al., 2019). Hence, in addition to its published role in modulating autophagosome-lysosome fusion through VAMP8 trafficking (Jean et al., 2015), RAB21 could also influence other steps of autophagy via its interaction with TMED10.

In this study, we validated the interaction between RAB21 and TMED10, a cargo adaptor, and showed the role of RAB21 in modulating TMED10 localization. Considering that RAB21 and TMED10 are involved in the regulation of autophagy and γ-secretase activity, respectively, and the fact that both of these pathways are associated with Alzheimer's disease, it would be interesting to further investigate the functional relationship between these two trafficking proteins in Alzheimer's disease. We believe that this study provides a framework for further studies on the association between the early endosomal RAB GTPase RAB21 and p24 family members.

## MATERIALS AND METHODS

### Cell culture

All cell lines were grown in Dulbecco's modified Eagle's medium (DMEM) supplemented with penicillin, streptomycin and 10% fetal bovine serum (Wisent) under 5% CO_2_ at 37°C. HeLa and HCT116 Flp-In/T-REx cells and RAB21 knockout cell populations were described previously in (Del Olmo et al., 2019).

### Generation of DNA constructs

PCDNA3-TMED10:3xHA was generated by amplifying TMED10 by PCR from HeLa cDNA generated using the Superscript III First-Strand synthesis kit. The TMED10 PCR fragment was ligated into pCDNA3-3xHA using the In-Fusion cloning kit (Clontech). The GFP:TMED10 plasmid was a kind gift of Robert Blum (Blum and Lepier, 2008). All constructs were validated by sequencing.

### Immunoprecipitations

3.5×10^6^ HCT116 or HeLa cells were plated in 100 mm dishes and grown for 24 h with 11 ng/ml of doxycycline to allow GFP:RAB21 induction. HCT116 cells were washed twice with cold PBS and lysed on ice for 20 min with 1 ml CoIP Buffer (1% IGEPAL CA-630, 1 mM EDTA, 150 mM NaCl, 0.1 mM EGTA, 25 mM Tris–HCl pH 7.4, 15 mM MgCl_2_, 2 mM Na_3_VO_4_, 10% glycerol, 2× protease inhibitors) per plate. HeLa cells were fixed for 15 min at room temperature with 0.5% formaldehyde with gentle rocking, following which formaldehyde was quenched for 5 min with 125 mM glycine at room temperature. Fixed HeLa cells were washed twice with cold PBS and lysed for 15 min at 4°C on a rotator with 1 ml of lysis buffer (1% IGEPAL CA-630, 1 mM EDTA, 150 mM NaCl, 25 mM Tris pH 7.4, 5% glycerol). For both HeLa and HCT116 cells, remaining membrane aggregates and DNA were removed by centrifugation at 16,000 ***g*** for 12 min at 4°C. Protein concentrations were determined using a BSA assay (Pierce), and immunoprecipitations were performed in individual tubes with equivalent quantities of proteins. 15 µl of GFP-Trap beads (ChromoTek) were used for individual IPs. For HCT116 cells, immunoprecipitations were performed on a rotator for 2.5 h at 4°C. Beads were then washed twice with CoIP buffer and twice again with CoIP buffer lacking IGEPAL CA-630. Immunoprecipitations in HeLa cells were carried out for 4 h on a rotator at 4°C. Following this incubation, beads were washed three times with lysis buffer. Finally, for both HCT116 and HeLa cells, excessive wash buffer was removed from the beads at the end of the immunoprecipitation protocol and 25 µl of 2× SDS loading buffer was added to each sample to elute proteins from beads.

### Pulldown assays

3.5×10^6^ HeLa cells were grown for 24 h in 100 mm plates, after which cells were lysed for 20 min on ice using 1 ml of MLB modified buffer (1% IGEPAL CA-630, 10% glycerol, 100 µM EGTA and 100 µM GTP, 25 mM HEPES, 150 mM NaCl, 20 mM MgCl_2_ and 1 mM sodium orthovanadate) supplemented with 2× protease inhibitors. Lysates were cleared by centrifugation at 16,000 ***g*** for 12 min at 4°C. GST and GST:RAB21 were purified following this (Jean et al., 2012). Prior to the pulldowns, GST and GST:RAB21 beads were washed three times in MLB modified buffer minus IGEPAL CA-630 and incubated on a rotator at 4°C for 20 min in MLB modified buffer lacking IGEPAL CA-630. Beads were further washed three times in complete MLB modified buffer and 900 µl of HeLa cell lysates was added to the beads for each pulldown, this was incubated for 1 h at 4°C on a rotator. Following this incubation, beads were washed three times with MLB modified buffer containing 0.2% IGEPAL CA-630. Protein were eluted with 30 µl of 2× SDS loading buffer.

### Immunoblots

For immunoblot analyses, 3×10^5^ parental HeLa cells and 4.5×10^5^ HeLa RAB21 KO cells were grown for 24 h in six-well plates. Cells were lysed with 200 µl of CoIP buffer as described above for immunoprecipitations. Lysates were quantified and the same amounts of proteins were used for analysis. Proteins were separated on 4–20% TGX precast gels (Bio-Rad) and transferred onto PVDF membranes (Millipore) using the trans-blot turbo system from Bio-Rad. Antibodies used for immunoblotting were anti-RAB21 (1:1000, Invitrogen #PA5-34404), anti-GFP (1:500, Santa Cruz #9996), anti-TMED10 (1:1000, Abcam #134948), anti-TMED2 (1:1000, Santa Cruz #376458), anti-GAPDH (1:8000, Cell Signaling #8884), anti-LC3B (1:1000, Cell Signaling #3868), anti-TGN38 (1:1000, Santa Cruz #166594), anti-Ubiquitin (1:1000, Cell Signaling 3933), anti-Vps35 (1:500, Santa Cruz #374372) and anti-rabbit and mouse HRP (1:10,000, Jackson Laboratories #115-035-144 and #115-035-146, respectively). Membranes were imaged on a Bio-Rad Chemidoc XR station following 5 min incubation with Luminata Forte (Millipore) or Clarity Max chemiluminescent substrates (Bio-Rad). On specific occasions, membranes were cut to allow probing with multiple antibodies simultaneously.

TMED10 stability experiments were performed by incubating cells in full media containing either 25 µg/ml cycloheximide or 10 µM MG132 for the indicated amount of time. For Bafilomycin A1 treatments, 0.2 µg/ml and 0.1 µg/ml were used for the 4 h and the 16 h time point, respectively. A lower concentration was used for the 16 h time point due to Baf A1 toxicity. Cells were lysed and proteins immunoblotted as described above.

### Immunofluorescence, colocalization and proximity ligation assay

A total of 20,000 wild-type HeLa or 30,000 RAB21 knockout cells were plated on glass coverslips (#1.5) in 24-wells plate and cultured overnight. The following day, pcDNA3-GFP:TMED10 or pcDNA3-TMED10:3xHA with or without pcDNA3-V5:RAB21 were transfected using Jetprime (Polyplus) following manufacturer's instructions. 24 h following transfection, cells were washed twice with 1× PBS and fixed for 15 min at room temperature with 250 µl of 4% paraformaldehyde in PBS. Cells were then washed three times 5 min each with 1× PBS. Fixed cells were blocked, and permeabilized for 60 min with 300 µl of 5% goat serum and 0.3% Triton X-100 in PBS. Cells were then incubated overnight in a humidified chamber at 4°C with primary antibody in 1× PBS containing 0.3% Triton X-100 and 1% BSA. The following day, primary antibodies were washed three times 5 min with 1× PBS. For immunofluorescence, cells were incubated for 1 h in a humidified chamber with secondary antibody at room temperature in the same buffer as the primary antibody. Secondary antibodies were washed three times for 5 min with 1× PBS at room temperature and cells were mounted in DAPI-containing mounting media (Sigma-Aldrich). For PLA, after having washed the primary antibodies, cells were incubated in a humidified chamber for 1 h at 37°C with Sigma probes (+) and (−) in the 1× Sigma dilution solution. Sigma probes were washed twice for 5 min with 1× wash buffer A. The ligation step was performed at 37°C for 30 min followed by two washes with buffer A. Amplification was performed for 100 min at 37°C in the dark. Cells were washed at room temperature twice for 10 min each with 1× wash buffer B and finally for 1 min in 0.01× wash buffer B and mounted in DAPI-containing mounting media (Sigma-Aldrich). Antibodies used for immunofluorescences were anti-GM130 (1:100, Cell Signaling #12480), anti-ciMPR (1:100, Bio-Rad #MCA2048T) and anti-TGN46 (1:100, Novus Biological #﻿NBP1-49643SS) and for PLA were anti-HA (1:1000, Cell Signaling #3724) and anti-V5 (1:5000, Sigma-Aldrich #V8012).

### Image analysis and statistics

All images were acquired on an Olympus FV1000 using a 63× 1.42NA plan Apo N objective or on a Zeiss LSM880 using a 40× 1.4NA plan Apo objective. Imaging settings were selected to minimize pixel saturation and to ensure proper Pearson correlation calculation. For figure preparation, all microscopy images were tresholded and cropped on Adobe Photoshop and assembled using Adobe Illustrator. All images were treated similarly, and only linear modifications were performed. The number of PLA puncta per cell was established using Cell Profiler. Briefly, a pipeline allowing transfected cell identification (using GFP signal from cotransfection of a small amount of pEGFP-C1 together with the other plasmids) was used and the number of PLA puncta per EGFP-positive cell was established using the relate and filter modules of Cell Profiler. Pearson correlations were also generated using Cell Profiler, with the distinction that cells were manually identified. Immunoblot data densitometry and image processing were performed using Image Lab (Bio-Rad). Immunoblot images were cropped and assembled using Photoshop and Illustrator respectively. For statistical analyses of images or immunoblots, every sample was first subjected to a normality test (if a sufficient number of values were present), following which either unpaired *t*-test, Mann–Whitney tests or one-sample *t*-tests were performed.

### Quantitative PCR analysis

Wild-type or RAB21 knockout cells were grown to 80% confluency in full media. CDNAs were prepared using the Maxima First Strand cDNA synthesis kit for RT-qPCR with dsDNAse (Thermo Fisher Scientific) following the manufacturer's instructions. Luna Universal qPCR Master Mix was used for amplification and the reactions were performed on a Roche LightCycler 96. Relative mRNA levels were calculated using the ΔΔCt method and normalized to GAPDH. TMED10, 2 and GAPDH primers were predesigned qPCR primers obtained from IDT.

## Supplementary Material

Supplementary information

## References

[BIO045336C1] Alanko J., Mai A., Jacquemet G., Schauer K., Kaukonen R., Saari M., Goud B. and Ivaska J. (2015). Integrin endosomal signalling suppresses anoikis. *Nat. Cell Biol.* 17, 1412-1421. 10.1038/ncb3250 26436690 PMC4890650

[BIO045336C2] Anantharaman V. and Aravind L. (2002). The GOLD domain, a novel protein module involved in Golgi function and secretion. *Genome Biol.* 3, research0023 10.1186/gb-2002-3-5-research0023 12049664 PMC115225

[BIO045336C3] Barr F. and Lambright D. G. (2010). Rab GEFs and GAPs. *Curr. Opin. Cell Biol.* 22, 461-470. 10.1016/j.ceb.2010.04.007 20466531 PMC2929657

[BIO045336C4] Blomen V. A., Májek P., Jae L. T., Bigenzahn J. W., Nieuwenhuis J., Staring J., Sacco R., van Diemen F. R., Olk N., Stukalov A. et al. (2015). Gene essentiality and synthetic lethality in haploid human cells. *Science* 350, 1092-1096. 10.1126/science.aac7557 26472760

[BIO045336C5] Blum R. and Lepier A. (2008). The luminal domain of p23 (Tmp21) plays a critical role in p23 cell surface trafficking. *Traffic* 9, 1530-1550. 10.1111/j.1600-0854.2008.00784.x 18627576

[BIO045336C6] Burgo A., Sotirakis E., Simmler M.-C., Verraes A., Chamot C., Simpson J. C., Lanzetti L., Proux-Gillardeaux V. and Galli T. (2009). Role of Varp, a Rab21 exchange factor and TI-VAMP/VAMP7 partner, in neurite growth. *EMBO Rep.* 10, 1117-1124. 10.1038/embor.2009.186 19745841 PMC2759737

[BIO045336C7] Burgo A., Proux-Gillardeaux V., Sotirakis E., Bun P., Casano A., Verraes A., Liem R. K. H., Formstecher E., Coppey-Moisan M. and Galli T. (2012). A molecular network for the transport of the TI-VAMP/VAMP7 vesicles from cell center to periphery. *Dev. Cell* 23, 166-180. 10.1016/j.devcel.2012.04.019 22705394

[BIO045336C8] Contreras I., Ortiz-Zapater E. and Aniento F. (2004). Sorting signals in the cytosolic tail of membrane proteins involved in the interaction with plant ARF1 and coatomer. *Plant J.* 38, 685-698. 10.1111/j.1365-313X.2004.02075.x 15125774

[BIO045336C9] Contreras F.-X., Ernst A. M., Haberkant P., Björkholm P., Lindahl E., Gönen B., Tischer C., Elofsson A., von Heijne G., Thiele C. et al. (2012). Molecular recognition of a single sphingolipid species by a protein's transmembrane domain. *Nature* 481, 525-529. 10.1038/nature10742 22230960

[BIO045336C10] Del Olmo T., Lauzier A., Normandin C., Larcher R., Lecours M., Jean D., Lessard L., Steinberg F., Boisvert F.-M. and Jean S. (2019). APEX2-mediated RAB proximity labeling identifies a role for RAB21 in clathrin-independent cargo sorting. *EMBO Rep.* 20, e47192 10.15252/embr.201847192 30610016 PMC6362359

[BIO045336C11] D'Souza-Schorey C. and Chavrier P. (2006). ARF proteins: roles in membrane traffic and beyond. *Nat. Rev. Mol. Cell Biol.* 7, 347-358. 10.1038/nrm1910 16633337

[BIO045336C12] Füllekrug J., Suganuma T., Tang B. L., Hong W., Storrie B. and Nilsson T. (1999). Localization and recycling of gp27 (hp24gamma3): complex formation with other p24 family members. *Mol. Biol. Cell* 10, 1939-1955. 10.1091/mbc.10.6.1939 10359607 PMC25391

[BIO045336C13] Gommel D., Orci L., Emig E. M., Hannah M. J., Ravazzola M., Nickel W., Helms J. B., Wieland F. T. and Sohn K. (1999). p24 and p23, the major transmembrane proteins of COPI-coated transport vesicles, form hetero-oligomeric complexes and cycle between the organelles of the early secretory pathway. *FEBS Lett.* 447, 179-185. 10.1016/S0014-5793(99)00246-X 10214941

[BIO045336C14] Grosshans B. L., Ortiz D. and Novick P. (2006). Rabs and their effectors: achieving specificity in membrane traffic. *Proc. Natl. Acad. Sci. USA* 103, 11821-11827. 10.1073/pnas.0601617103 16882731 PMC1567661

[BIO045336C15] Hein M. Y., Hubner N. C., Poser I., Cox J., Nagaraj N., Toyoda Y., Gak I. A., Weisswange I., Mansfeld J., Buchholz F. et al. (2015). A human interactome in three quantitative dimensions organized by stoichiometries and abundances. *Cell* 163, 712-723. 10.1016/j.cell.2015.09.053 26496610

[BIO045336C16] Hutagalung A. H. and Novick P. J. (2011). Role of Rab GTPases in membrane traffic and cell physiology. *Physiol. Rev.* 91, 119-149. 10.1152/physrev.00059.2009 21248164 PMC3710122

[BIO045336C17] Jean S. and Kiger A. A. (2012). Coordination between RAB GTPase and phosphoinositide regulation and functions. *Nat. Rev. Mol. Cell Biol.* 13, 463-470. 10.1038/nrm3379 22722608

[BIO045336C18] Jean S., Cox S., Schmidt E. J., Robinson F. L. and Kiger A. (2012). Sbf/MTMR13 coordinates PI(3)P and Rab21 regulation in endocytic control of cellular remodeling. *Mol. Biol. Cell* 23, 2723-2740. 10.1091/mbc.e12-05-0375 22648168 PMC3395661

[BIO045336C19] Jean S., Cox S., Nassari S. and Kiger A. A. (2015). Starvation-induced MTMR13 and RAB21 activity regulates VAMP8 to promote autophagosome-lysosome fusion. *EMBO Rep.* 16, 297-311. 10.15252/embr.201439464 25648148 PMC4364869

[BIO045336C20] Jun Y.-S. and Ahn K.-S. (2011). Tmp21, a novel MHC-I interacting protein, preferentially binds to β_2_-microglobulin-free MHC-I heavy chains. *BMB Rep.* 44, 369-374. 10.5483/BMBRep.2011.44.6.369 21699748

[BIO045336C21] Lee S.-Y., Kang M.-G., Shin S., Kwak C., Kwon T., Seo J. K., Kim J.-S. and Rhee H.-W. (2017). Architecture mapping of the inner mitochondrial membrane proteome by chemical tools in live cells. *J. Am. Chem. Soc.* 139, 3651-3662. 10.1021/jacs.6b10418 28156110

[BIO045336C22] Liu S., Bromley-Brits K., Xia K., Mittelholtz J., Wang R. and Song W. (2008). TMP21 degradation is mediated by the ubiquitin-proteasome pathway. *Eur. J. Neurosci.* 28, 1980-1988. 10.1111/j.1460-9568.2008.06497.x 19046380

[BIO045336C23] Luo W., Wang Y. and Reiser G. (2007). p24A, a type I transmembrane protein, controls ARF1-dependent resensitization of protease-activated receptor-2 by influence on receptor trafficking. *J. Biol. Chem.* 282, 30246-30255. 10.1074/jbc.M703205200 17693410

[BIO045336C24] Majoul I., Straub M., Hell S. W., Duden R. and Soeling H.-D. (2001). KDEL-cargo regulates interactions between proteins involved in COPI vesicle traffic: measurements in living cells using FRET. *Dev. Cell* 1, 139-153. 10.1016/S1534-5807(01)00004-1 11703931

[BIO045336C25] Montesinos J. C., Sturm S., Langhans M., Hillmer S., Marcote M. J., Robinson D. G. and Aniento F. (2012). Coupled transport of Arabidopsis p24 proteins at the ER-Golgi interface. *J. Exp. Bot.* 63, 4243-4261. 10.1093/jxb/ers112 22577184 PMC3398454

[BIO045336C26] Pardossi-Piquard R., Böhm C., Chen F., Kanemoto S., Checler F., Schmitt-Ulms G., St George-Hyslop P. and Fraser P. E. (2009). TMP21 transmembrane domain regulates γ-secretase cleavage. *J. Biol. Chem.* 284, 28634-28641. 10.1074/jbc.M109.059345 19710022 PMC2781407

[BIO045336C27] Pastor-Cantizano N., Montesinos J. C., Bernat-Silvestre C., Marcote M. J. and Aniento F. (2016). p24 family proteins: key players in the regulation of trafficking along the secretory pathway. *Protoplasma* 253, 967-985. 10.1007/s00709-015-0858-6 26224213

[BIO045336C28] Pellinen T., Arjonen A., Vuoriluoto K., Kallio K., Fransen J. A. M. and Ivaska J. (2006). Small GTPase Rab21 regulates cell adhesion and controls endosomal traffic of beta1-integrins. *J. Cell Biol.* 173, 767-780. 10.1083/jcb.200509019 16754960 PMC2063892

[BIO045336C29] Popoff V., Adolf F., Brügger B. and Wieland F. (2011). COPI budding within the Golgi stack. *Cold Spring Harb. Perspect. Biol.* 3, a005231 10.1101/cshperspect.a005231 21844168 PMC3220356

[BIO045336C30] Rojas A. M., Fuentes G., Rausell A. and Valencia A. (2012). Evolution: the Ras protein superfamily: evolutionary tree and role of conserved amino acids. *J. Cell Biol.* 196, 189-201. 10.1083/jcb.201103008 22270915 PMC3265948

[BIO045336C31] Shin J. H., Park S. J., Jo D. S., Park N. Y., Kim J. B., Bae J.-E., Jo Y. K., Hwang J. J., Lee J.-A., Jo D.-G. et al. (2019). Down-regulated TMED10 in Alzheimer disease induces autophagy via ATG4B activation. *Autophagy* 15, 1495-1505. 10.1080/15548627.2019.1586249 30821607 PMC6693468

[BIO045336C32] Simpson J. C., Griffiths G., Wessling-Resnick M., Fransen J. A. M., Bennett H. and Jones A. T. (2004). A role for the small GTPase Rab21 in the early endocytic pathway. *J. Cell Sci.* 117, 6297-6311. 10.1242/jcs.01560 15561770

[BIO045336C33] Stenmark H. (2009). Rab GTPases as coordinators of vesicle traffic. *Nat. Rev. Mol. cell Biol.* 10, 513-525. 10.1038/nrm2728 19603039

[BIO045336C34] Sun Z., Xie Y., Chen Y., Yang Q., Quan Z., Dai R. and Qing H. (2017). Rab21, a novel PS1 interactor, regulates γ-secretase activity via PS1 subcellular distribution. *Mol. Neurobiol.* 55, 3841-3855. 10.1007/s12035-017-0606-3 28547526

[BIO045336C35] Theiler R., Fujita M., Nagae M., Yamaguchi Y., Maeda Y. and Kinoshita T. (2014). The α-helical region in p24γ2 subunit of p24 protein cargo receptor is pivotal for the recognition and transport of glycosylphosphatidylinositol-anchored proteins. *J. Biol. Chem.* 289, 16835-16843. 10.1074/jbc.M114.568311 24778190 PMC4059126

[BIO045336C36] Vicinanza M., D'Angelo G., Di Campli A. and De Matteis M. A. (2008). Function and dysfunction of the PI system in membrane trafficking. *EMBO J.* 27, 2457-2470. 10.1038/emboj.2008.169 18784754 PMC2536629

[BIO045336C37] Wang D., Lou J., Ouyang C., Chen W., Liu Y., Liu X., Cao X., Wang J. and Lu L. (2010). Ras-related protein Rab10 facilitates TLR4 signaling by promoting replenishment of TLR4 onto the plasma membrane. *Proc. Natl. Acad. Sci. USA* 107, 13806-13811. 10.1073/pnas.1009428107 20643919 PMC2922283

[BIO045336C38] Wang H. B., Xiao L. and Kazanietz M. G. (2011). p23/Tmp21 associates with protein kinase Cδ (PKCδ) and modulates its apoptotic function. *J. Biol. Chem.* 286, 15821-15831. 10.1074/jbc.M111.227991 21454541 PMC3091192

[BIO045336C39] Yang X., Zhang Y., Li S., Liu C., Jin Z., Wang Y., Ren F. and Chang Z. (2012). Rab21 attenuates EGF-mediated MAPK signaling through enhancing EGFR internalization and degradation. *Biochem. Biophys. Res. Commun.* 421, 651-657. 10.1016/j.bbrc.2012.04.049 22525675

[BIO045336C40] Zavodszky E. and Hegde R. S. (2019). Misfolded GPI-anchored proteins are escorted through the secretory pathway by ER-derived factors. *eLife* 8, e46740 10.7554/eLife.46740 31094677 PMC6541436

[BIO045336C41] Zhao P., Metcalf M. and Bunnett N. W. (2014). Biased signaling of protease-activated receptors. *Front. Endocrinol. (Lausanne).* 5, 67 10.3389/fendo.2014.00067 24860547 PMC4026716

