# Supplementary material for: RAB21 interacts with TMED10 and modulates its localization and abundance
Source: Biol Open. 2019 Aug 27;8(9):bio045336. doi: 10.1242/bio.045336 (PMC6777364; doi:10.1242/bio.045336)
Supplement: Supplementary information [file biolopen-8-045336-s1.pdf]

Supplementary information

A

	HeLa			HCT116		
	SILAC ratios (fold over control)					
	WT	Q78L	T33N	WT	Q78L	T33N
TMED10	**3.5**	**2.6**	**1.8**	**3.1**	**7.7**	**1.8**
TMED9	**2.8**	**2.0**	**1.3**	**2.9**	**4.1**	**1.2**

B

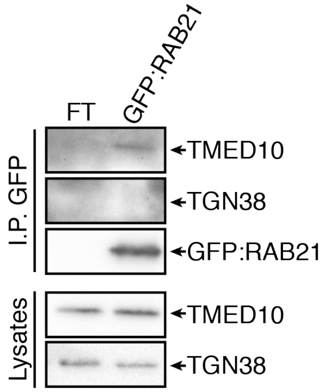

C

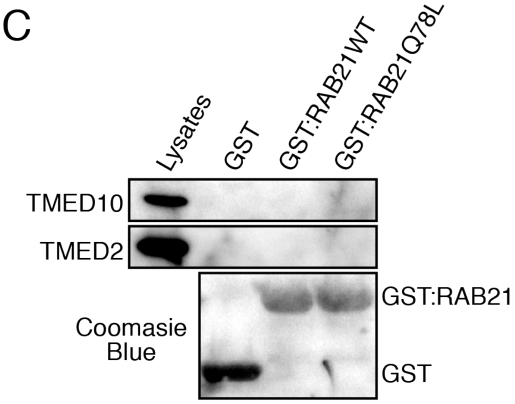

D

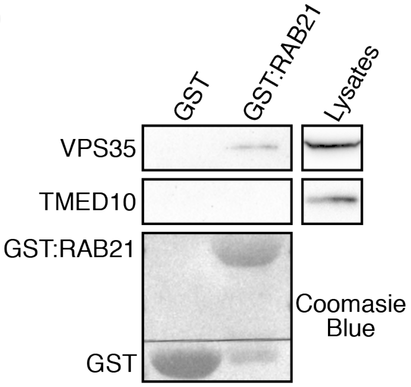

Figure S1. Indirect interaction between RAB21 and TMED10

(A) SILAC enrichment ratios of GFP:RAB21 variants over the FT control in HeLa and HCT116 cells, data from (Del Olmo et al., 2019). (B) Western blotting showing GFP-trap immunoprecipitation in HeLa cells. Endogenous TMED10 and TGN38 were blotted. A specific enrichment with GFP:RAB21 compared to FT control was only observed with TMED10. Lysates represents 2% of input and n=3 independent experiments. (C) Western blotting showing GST:RAB21, GST:RAB21-Q78L pull-downs and GST only as control. TMED10 and TMED2 were blotted and showed no specific enrichment with neither RAB21 or RAB21-Q78L compared to control. Gels were stained with coomasie blue to compare purified protein levels in all conditions, n=2 independent experiments. (D) Western blotting showing GST:RAB21 loaded with

Biology Open • Supplementary information

GTP, pull-downs and GST only as control. TMED10 and VPS35 were blotted. Only VPS35 showed a specific enrichment with GST:RAB21. Gels were stained with coomasie blue to compare purified protein levels in all conditions, n=3 independent experiments.

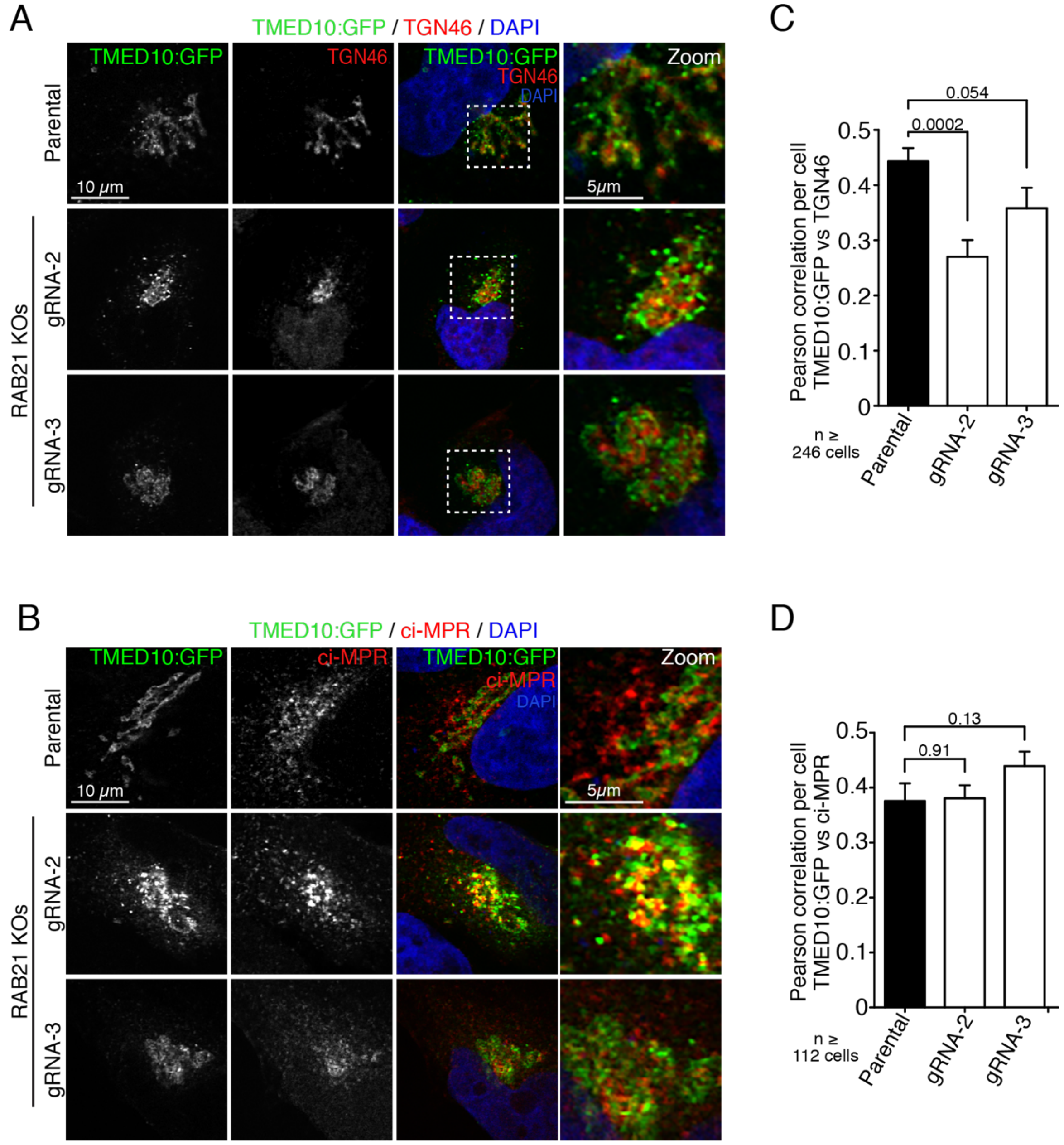

Figure S2. TMED10 localization at *trans*-Golgi is modulated by RAB21.

Biology Open • Supplementary information

(A) TMED10:GFP colocalization with endogenous TGN46 in parental and RAB21 knockout HeLa cells. TMED10:GFP is stained in green, TGN46 in red and nucleus in blue. Scale bar, 10 μm. Dotted squares are magnified, scale bar 5 μm, n=3 independent experiments. (B) Same as in (A) immunofluorescence showing colocalization between endogenous ci-MPR and TMED10:GFP, n=3 independent experiments. (C) Quantification of TMED10 and TGN46 colocalization showed in (A). Histograms represent average Pearson correlation per cell, error bars are SEM. (D) Same as in (C) quantification of TMED10 and ci-MPR colocalization showed in (B), error bars are SEM. Mann-Whitney tests were used for statistical analysis in C and D.

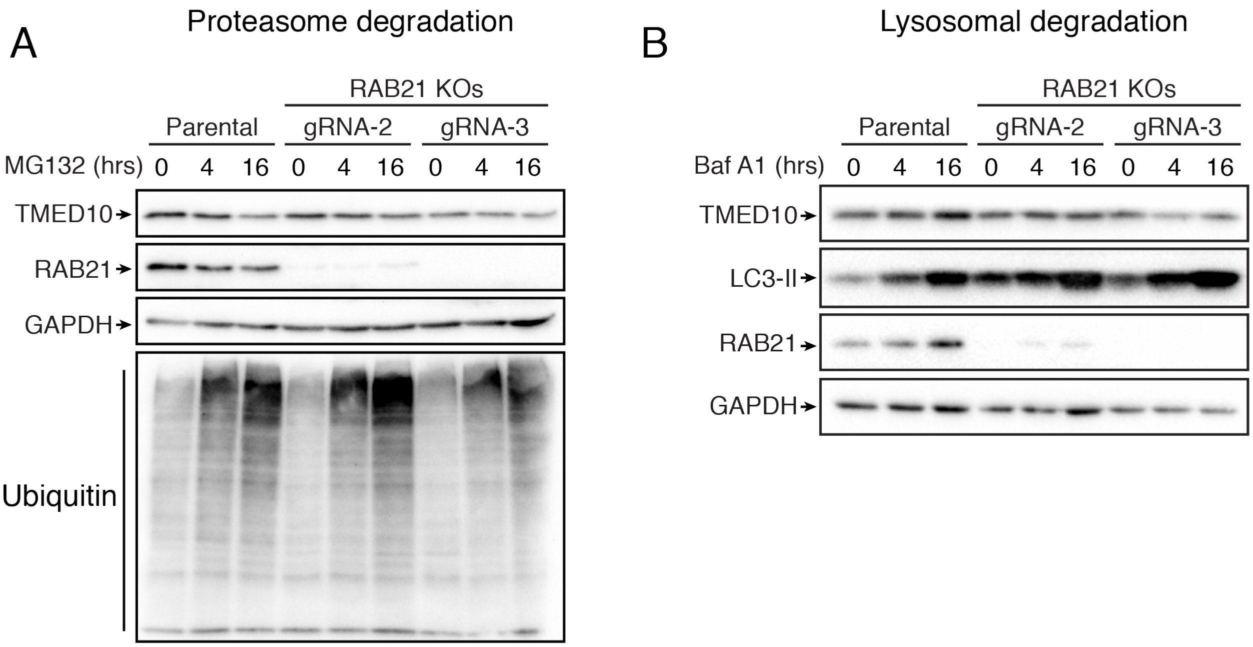

Figure S3. TMED10 stability is not affected by blocking proteasomal or lysosomal functions. Proteasome inhibition does not stabilize TMED10 over a 16h treatment. (A) A MG132 chase was performed to monitor TMED10 stability in parental and RAB21 gRNA-2 and -3 KO HeLa cell lines, n=3 independent experiments. Endogenous TMED10, RAB21, GAPDH and Ubiquitin were

Biology Open • Supplementary information

assessed through western blotting. Note that the ubiquitin WB was performed on a different gel loaded with the same amount of proteins as for the GAPDH, RAB21 and TMED10 immunoblots, and was used to confirm the efficiency of the MG132 treatment. (B) A Bafilomycin A1 chase was performed to monitor TMED10 stability in parental and RAB21 gRNA-2 and -3 KO HeLa cell lines, n=3 independent experiments. Endogenous TMED10, RAB21, LC3-II and GAPDH were assessed through western blotting. The accumulation of LC3-II at 4h and 16h following BafA1 treatment confirms that lysosomal functions were affected.
